# NIR-activated adipose-targeted nanocarrier drives local browning and metabolic restoration

**DOI:** 10.1093/rb/rbag052

**Published:** 2026-03-09

**Authors:** Ying Li, Enze Liu, Pei Wang, Jiayi Liu, Weijie Jin, Zhen Gao, Jiayingzi Wu, Xiansong Wang

**Affiliations:** Department of Plastic and Reconstructive Surgery, Shanghai Key Laboratory of Tissue Engineering, Shanghai Ninth People’s Hospital, Shanghai Jiao Tong University School of Medicine, Shanghai 200011, China; Department of Plastic and Reconstructive Surgery, Shanghai Key Laboratory of Tissue Engineering, Shanghai Ninth People’s Hospital, Shanghai Jiao Tong University School of Medicine, Shanghai 200011, China; Department of Plastic and Reconstructive Surgery, Shanghai Key Laboratory of Tissue Engineering, Shanghai Ninth People’s Hospital, Shanghai Jiao Tong University School of Medicine, Shanghai 200011, China; Shenghua Zizhu Academy, Shanghai 200241, China; Division of Infection and Immunity, Faculty of Medical Science, University College London, London NW3 2PP, United Kingdom; Department of Plastic and Reconstructive Surgery, Shanghai Key Laboratory of Tissue Engineering, Shanghai Ninth People’s Hospital, Shanghai Jiao Tong University School of Medicine, Shanghai 200011, China; Guangdong Key Laboratory for Biomedical Measurements and Ultrasound Imaging, School of Biomedical Engineering, Medical School, Shenzhen University, Shenzhen 518055, China; Department of Plastic and Reconstructive Surgery, Shanghai Key Laboratory of Tissue Engineering, Shanghai Ninth People’s Hospital, Shanghai Jiao Tong University School of Medicine, Shanghai 200011, China

**Keywords:** anti-obesity, mesoporous polydopamine, photothermal browning therapy, resveratrol

## Abstract

The escalating global prevalence of obesity and its associated metabolic disorders now represents one of the most urgent public health crises. Despite the pressing need for safe and effective anti-obesity interventions, current therapeutic options are often limited by suboptimal efficacy and various side effects. Herein, we present a microneedle (MN)-based drug delivery platform, termed RES@hmPDA-Dpep/MN, for photothermal browning therapy (PBT) to combat obesity. This system integrates hemispherical mesoporous polydopamine (hmPDA) nanoparticles with resveratrol (RES) and an adipose-homing peptide (Dpep) for localized PBT. Under identical conditions, hmPDA maintains photothermal performance comparable to spherical mPDA, while exhibiting advantageous drug-loading behavior, particularly under higher drug-feeding conditions. In addition, the platform supports localized delivery with near-infrared-triggered, on-demand RES release and thermogenic activation. *In vivo* studies demonstrate that the RES@hmPDA-Dpep/MN system effectively suppresses white adipose tissue expansion, reduces body weight by 16.1 ± 2.5%, and significantly improves obesity-associated metabolic dysfunctions, including hyperlipidemia and hepatic steatosis. Mechanistic analyses reveal marked upregulation of uncoupling protein 1 (UCP1) and other key browning genes. Furthermore, the nanoplatform shows favorable *in vitro* biocompatibility and hemocompatibility, supporting its potential for further translational evaluation. Collectively, this PBT strategy may serve as a potential localized intervention for fat reduction and systemic metabolic improvement in the management of obesity and related metabolic disorders.

## Graphical abstract

**Graphical Abstract:**
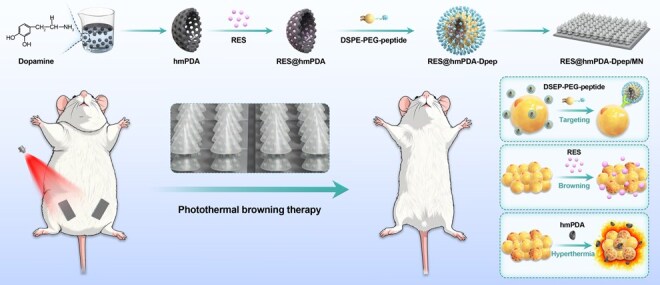

## Introduction

The global prevalence of obesity has risen dramatically over the past few decades, accompanied by a notable decrease in the age of onset [1, 2]. This epidemic is now recognized as a major public health crisis due to its substantial financial burden and its association with a wide range of comorbidities, including metabolic syndrome, cardiovascular diseases and type 2 diabetes [3]. Current therapeutic strategies for obesity primarily comprise lifestyle modification, pharmacotherapy and bariatric surgery. Although lifestyle intervention is recommended as the first-line treatment, long-term adherence remains challenging, and limits its effectiveness in the general population [4]. Pharmacological agents often target appetite regulation via central nervous system modulation, but individual variability in response and the occurrence of gastrointestinal side effects constrain their widespread clinical use. While bariatric surgery can achieve substantial weight loss, it carries risks such as nutritional deficiencies and cholelithiasis [5]. Therefore, there remains an urgent need for safe, effective and widely accessible anti-obesity therapies.

In recent years, photothermal therapy (PTT) has emerged as a promising non-invasive strategy for localized fat reduction. Traditional PTT typically operates at high temperatures (>46°C), which leads to lipolysis of lipid droplets and directly induces adipocyte death [6, 7]. However, it may cause adverse effects, including lipotoxicity, inflammation and damage to surrounding tissues in the treated area [8]. To mitigate these adverse effects, mild PTT (mPTT), typically within 40–42°C, has been developed [9, 10]. Although mPTT has achieved success in fat reduction, its impact on elevating metabolic health and alleviating obesity-related complications remains limited. Recent studies have revealed the broader metabolic roles of beige and brown adipocytes, including their ability to regulate systemic energy balance [11, 12]. Accordingly, this has led to the emergence of photothermal browning therapy (PBT), which combines PTT with “browning agents” [13], compounds capable of inducing conversion of white adipocytes into beige or brown adipocytes [14–16]. However, most browning agents exert anti-obesity effects through mechanisms that do not synergize effectively with hyperthermia, limiting the combined efficacy of PBT. Recently, Li *et al.* demonstrated that beige adipocytes respond to mild hyperthermia (∼41°C) by upregulating heat shock factor 1 (HSF1), thereby enhancing thermogenesis [17]. Furthermore, local heating in the supraclavicular region, where beige fat is typically located, increases heat production in adults. These findings suggest that beige adipocytes are thermosensitive and could be selectively activated by hyperthermia alone. Nonetheless, the limited distribution and abundance of endogenous beige fat pose a significant barrier to the broader application of this strategy.

To effectively promote the browning of white adipose tissue (WAT), the identification and development of suitable white-to-brown adipose tissue conversion agents are critical. With growing insights into the underlying thermogenic mechanisms, multiple factors must be considered when selecting candidate agents, including efficacy, safety and mechanism of action. The transformation of energy-storing WAT into energy-dissipating adipose tissue can be driven by endogenous, pharmacological or nutritional stimuli. However, the undesirable side effects associated with endogenous and pharmacological ingredients have shifted the attention to nutritional stimuli, such as dietary supplements and plant products, which offer safer alternatives. Among these phytochemicals, which are naturally occurring bioactive compounds found in plants, have attracted increasing interest due to their favorable safety profiles and metabolic benefits. Several phytochemicals have demonstrated remarkable anti-adipogenic activity as well as the ability to enhance thermogenesis [18]. Resveratrol (RES), a natural polyphenolic compound extracted from grapes and berries, offers multiple pharmacological benefits, including cardioprotective, antioxidant, antitumor as well as the ability to stimulate WAT browning by activating the AMPK pathway [19, 20]. Intriguingly, previous studies have reported that RES can activate HSF1 in a direct or indirect way [21, 22]. These findings highlight the potential of RES as an ideal browning agent candidate in PBT, given its ability to both increase the amount of induced beige adipocytes and amplify the effect of thermogenesis.

Conventional drug delivery strategies often suffer from poor tissue specificity, leading to nonspecific biodistribution, suboptimal therapeutic efficacy and increased systemic toxicity. Likewise, traditional transdermal delivery is limited by the skin’s stratum corneum barrier, resulting in low permeability and restricted drug absorption. To overcome these limitations, nanoparticle-based systems provide versatile platforms with tunable surface modifications and controlled drug release profiles, thereby improving targeting efficiency and pharmacokinetics. In parallel, microneedle (MN) technology enables minimally invasive, localized delivery across the skin barrier, offering enhanced bioavailability and patient compliance. The integration of functionalized nanoparticles with MNs holds great promise for site-specific drug accumulation and improved therapeutic outcomes in metabolic disorders. Thus, for efficient delivery of RES to adipose tissue, an MN-based transdermal delivery system was employed in this study. MNs provide a minimally invasive and patient-friendly route for delivering therapeutics into subcutaneous fat. To further enhance targeting specificity, the nanoparticles were functionalized with a modified adipose-homing peptide (AHP) [23], the peptide motif (sequence CKGGRAKDC) specifically binds to prohibitin (PHB)—a receptor highly expressed on the vasculature and mature adipocytes in WAT [24].

Herein, we report the design and evaluation of a novel MN patch loaded with RES-encapsulated hemispherical mesoporous polydopamine nanoparticles conjugated with the targeting peptide (RES@hmPDA-Dpep/MN). The hemispherical mesoporous polydopamine was developed instead of traditional spherical morphology to serve as both a photothermal convertor and a drug carrier, enabling heat generation and improved solubility of RES. RES functions as both a white-to-brown convertor and an amplifier of hyperthermia, while the modified AHP serves as a targeting strategy guiding the nanoparticles to the WAT. The MN patch provided a flexible approach for penetrating the subcutaneous fat layer in an optional position. The combination of photothermal stimulation, targeted drug delivery and RES-induced thermogenic activation enables a synergistic anti-obesity effect with minimal off-target toxicity. Experiments were conducted to evaluate the performance of the RES@hmPDA-Dpep/MN in anti-obesity treatment.

## Materials and methods

### Chemicals and materials

Insulin, dexamethasone and 3-isobutyl-1-methylxanthine (IBMX) were purchased from Shanghai Biyuntian Biotechnology Co., Ltd. (Shanghai, China). DMEM high-glucose medium, fetal bovine serum (FBS) and trypsin-containing digest with EDTA were purchased from Gibco (New York, NY, USA). The modified peptide (DSPE-PEG-CKGGRAKDC) was provided by Ruixi Biological Technology (Xi’an) Ltd. RES, 1,3,5-trimethylbenzene (CAS: 108-67-8), Dopamine hydrochloride (CAS: 62-31-7), Pluronic^®^ F-127 (CAS: 9003-11-6) were purchased from Aladdin (Shanghai, China). γ-PGA (Mw = 1000 kDa) was purchased from Sai Taisi Biological Technology Co., Ltd. (China). All other reagents and chemicals were of analytical grade.

### Synthesis of RES@hmPDA-Dpep

MPDA nanoparticles were prepared as previously described with a little modification [25]. Briefly, 1 g PF127 and 1.6 mL 1,3,5-trimethylbenzene were added to a mixture solution (50-mL ddH_2_O and 50-mL ethanol) in a Florence flask, and stirred for 10 min, then sonicated for 15 min to obtain a uniform milky white solution. Next, add 1.5 g dopamine hydrochloride into the solution and stir for 30 min, then add 4.5 mL ammonium hydroxide and stir with an open lid for 2 h. Centrifuge at 13 000 rpm for 15 min and the product was obtained after washing twice with 50% ethanol.

Owing to π–π stacking interactions, RES@hmPDA was made by a physical adsorption method. Briefly, 10-mg hemispherical mesoporous polydopamine (hmPDA) powder was added to 1 mg mL^−1^ RES (in PBS), stirred for 48 h in an ice bath. Washed twice with PBS and conducted vacuum freeze-drying to finally acquire RES@hmPDA.

RES@hmPDA-Dpep was obtained by RES@hmPDA coating with DSPE-PEG-CKGGRAKDC. The peptide-modified DSPE-PEG (4 mg) was dissolved in chloroform (4 mL), 20 mg RES@hmPDA nanoparticles dispersed in chloroform were added, and mixed for 12 h. The solvent was removed with a rotary evaporator. The RES@hmPDA-Dpep/MN was dispersed in water to hydrate, then transferred into a dialysis bag for 48 h to remove excess DSPE-PEG-CKGGRAKDC.

### Characterization of the nanocomplex RES@hmPDA-Dpep

The morphology of hmPDA, RES@hmPDA and RES@hmPDA-Dpep was observed by transmission electron microscope (TEM) and scanning electron microscopy (SEM). The particle size was calculated by Fiji according to the SEM images. The zeta potential and diameter of hmPDA, RES@hmPDA and RES@hmPDA-Dpep were evaluated by dynamic light scattering (DLS). The structural properties of the synthesized nanoparticles were analyzed using Brunauer–Emmett–Teller (BET) measurements. The UV–visible absorption spectrum of each sample was tested and recorded by a UV spectrophotometer (Shimadzu UV-3600i Plus, Kyoto, Japan).

The loading capacity (LC) was analyzed by a microplate reader with a standard curve at 304 nm. The calculation formula is as follows: (total RES − suspension RES)/total RES * 100%.

### Construction and photothermal property of RES@hmPDA-Dpep/MN

MN patches were established according to our previous experience [25]. Fabrication of RES@hmPDA-Dpep/MN: every 10 mg of all kinds of nanoparticles or 5 mg RES was first uniformly scattered into 600-μL ddH_2_O. Precursor hydrogel was prepared by completely dissolving 350-mg γ-PGA into the mixture solution. Afterwards, 200-μL mixture was picked into an MN model, centrifuged at 3750 rpm to fill the needle tips, then the model was put into a drying oven at 40°C overnight and demolded to obtain one patch, stored in a cool, desiccated environment. Likewise, Blank MN patches consisting of γ-PGA alone were fabricated following the same procedure.

Thermo-properties were conducted by a near-infrared (NIR) laser and recorded by a thermal imager. RES@hmPDA-Dpep solutions of different concentrations (0.5, 1 and 1.5 mg mL^−1^) were suspended in ultrapure water, and MNs with 1.5 mg mL^−1^ nanoparticles were placed on porcine tissue. All of them were irradiated with an 808-nm NIR light at different intensities for a certain period.

### Compression testing of MN patches

The compressive mechanical strength of the dissolving MN patches was evaluated using a mechanical testing system. MN patches were mounted on a rigid base with the needle tips facing upward, and a flat stainless-steel plate was aligned perpendicular to the MN array. Compression was performed at a constant displacement rate until the force–displacement curve exhibited an apparent plateau.

### *In vitro* drug release

A single MN patch was put in a 15-mL centrifuge tube containing 10-mL simulated body fluid (SBF), then placed in a horizontal shaker (37°C, 110 rpm). Collect 1-mL supernatant at each time point and replenish 1-mL fresh SBF every time. The 808-nm laser was applied for 5 min before sample collection. The concentration of released RES was calculated by a microplate reader with a standard curve.

### Biocompatibility of nanoparticles

Saline and pure water were used as the negative and positive controls, respectively. The hemolysis test was performed to detect the biocompatibility of MNs, and the rate was calculated as follows:


$$\text{Hemolytic}\;\text{rate}\left(\%\right)=\left(A_{\text{Sample}}-A_{\text{Saline}}\right)/\left(A_{\text{Water}}-A_{\text{Saline}}\right)\times 100\%$$

The influence of cell viability in different concentrations of RES against differentiated 3T3-L1 cells was determined by the Cell Counting Kit-8 (Dojindo) assay. Cytotoxicity toward differentiated 3T3-L1 cells was further evaluated using Calcein-AM/PI Double Staining Kit (Dojindo).

### *In vitro* cellular uptake of RES@hmPDA-Dpep

The RES, hmPDA, RES@hmPDA, RES@hmPDA-Dpep were labeled with Rhodamine, then incubated with differentiated and undifferentiated 3T3-L1 cells for 24 h. The cells were then stained with PHB antibody and DAPI. For immunofluorescence analysis, the following antibodies were used: CoraLite488-conjugated Goat Anti-Rabbit IgG(H + L) (Proteintech, SA00013-2; 1:500), Prohibitin Recombinant Rabbit Monoclonal Antibody (SR46-02) (HUABIO, Zhejiang, China), Rhodamine (Sigma-Aldrich (Shanghai) Trading Co.Ltd.). The immunofluorescence images were captured by a THUNDER Imager (Leica).

### Skin cross-section fluorescence imaging

For immunofluorescence staining, sections were permeabilized with 0.1% Triton X-100 in PBS, blocked with 5% BSA (or normal serum) for 1 h, and incubated overnight at 4°C with a primary antibody against perilipin (Proteintech, anti-Perilipin-1, 1:200). After washing, sections were incubated with an Alexa Fluor-conjugated secondary antibody (Proteintech, Alexa Fluor 488, 1:500) for 1 h at room temperature. Nuclei were counterstained with DAPI. Fluorescence images were acquired using a confocal fluorescence microscope.

### *In vitro* anti-adipogenic ability

The anti-adipogenic ability was estimated by Oil-Red-O staining, and mature adipocytes were induced from 3T3-L1 cells according to a cocktail method. Briefly, 3T3-L1 cells were seeded in a six-well plate, the inducible culture medium (10% FBS, 1% PS, 10 μg mL^−1^ insulin, 1 μM dexamethasone, 0.5 mM IBMX) and maintenance medium (10% FBS, 1% PS, 10 μg mL^−1^ insulin) were applied in sequence after a fully confluence was reached. Cells were then treated with 100 μL of leaching agent from different groups for 24 h, then the supernatant was discarded, cells were washed twice with PBS, then fixed with 10% PFA for 20 min at room temperature. After thorough washing, cells were stained with Oil-Red-O solution for 30 min. The results were observed under an optical microscope.

### *In vivo* anti-obesity ability of RES@hmPDA-Dpep/MNs

Diet-induced obesity in mice was established by continuous feeding with a high-fat diet for at least 8 weeks. The initial weight of obese mice was set at approximately 35 g. MN patches (containing 1.5 mg mL^−1^ RES@hmPDA-Dpep in pre-solution) were applied under gaseous anesthesia every 3 days. Body weight and food intake were recorded at each treatment. After six treatments, the inguinal adipose tissue, epididymal adipose tissue and liver were harvested, weighed and stored in 10% PFA for subsequent histological analysis. All experimental procedures were carried out in accordance with the institutional guidelines of laboratory animal care and use and were approved by the Animal Care and Use Committee of Shanghai Ninth People’s Hospital (approval number: SH9H-2025-A546-SB).

### RNA isolation and quantitative RT-PCR

Total RNA from adipose tissue biopsies and 3T3-L1 cells was isolated with RNA Purification Kit (EZB), according to the manufacturer’s recommendations. Primer sequences were obtained from the literature and listed as follows: Gapdh (F: AGGTCGGTGTGAACGGATTTG, R: TGTAGACCATGTAGTTGAGGTCA); Ucp1 (F: GGCCCTTGTAAACAACAAAATAC, R: GGCAACAAGAGCTGACAGTAAAT); Hsf1 (F: GCACAACAACATGGCTAGCT, R: TGACACTGTCCTGGCGTATT); Trpv1(F: CCGGCTTTTTGGGAAGGGT, R: GAGACAGGTAGGTCCATCCAC); Prdm16 (F: CCCCACATTCCGCTGTGAT, R: CTCGCAATCCTTGCACTCA); Hnrnpa2b1 (F: CAGGGTAGTTGAGCCAAAACG, R: TTCCAGACTGCCTATCGGTAA); Tbx1 (F: CTGTGGGACGAGTTCAATCAG, R: TTGTCATCTACGGGCACAAAG). RT-qPCR was conducted using an RT Master Mix (Takara) guided by the provided protocols. Target mRNA was calculated using the 2^−ΔΔCt^ method.

### Histological assessment

The inguinal and epididymal adipose tissues of each mouse were completely separated and further immersed in 4% paraformaldehyde for 24 h. The samples were then embedded in wax blocks and cut into 4-µm-thick sections for histological staining. The morphology of inguinal and epididymal adipose tissue after treatment was detected by hematoxylin and eosin (H&E) staining. The browning degree of tissue was detected by immunohistochemical staining of UCP-1 (beige/brown adipocyte marker).

### Evaluation of serologic parameters

Blood samples were collected from the retro-orbital sinus after anesthesia and stored in an anticoagulant tube. Centrifuge at 1200 rpm for 10 min, the supernatant was transferred to a new Eppendorf tube and experienced another centrifugation of 13 000 rpm, 10 min. Serum lipid parameters [low-density lipoprotein cholesterol (LDL-C), total cholesterol, triglycerides (TG)] were detected by a fully automatic biochemical analyzer.

### Statistical analysis

**Figure 1:**
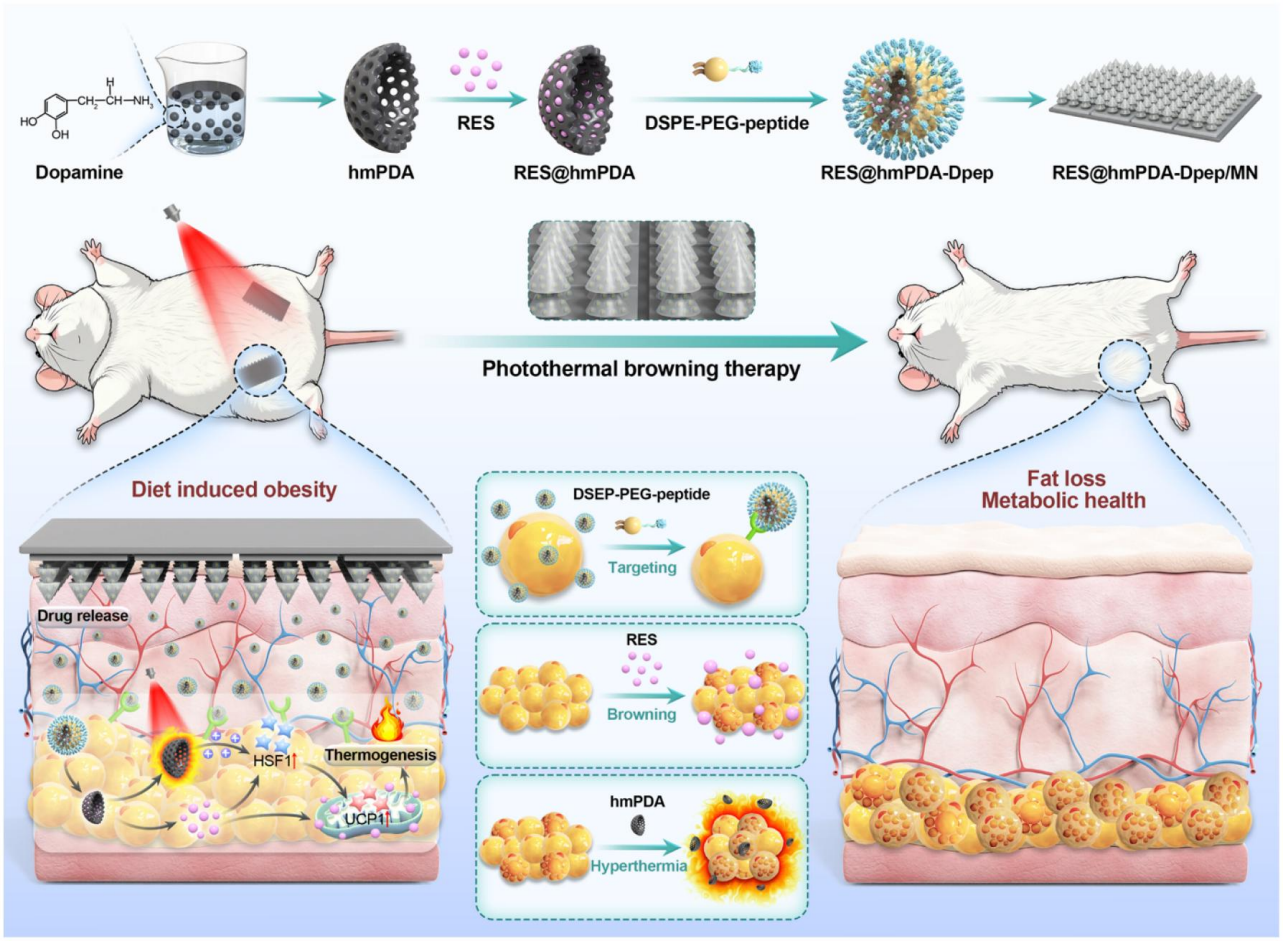
Schematic illustration of the photothermal browning therapy mediated by multicomponent hemispherical mesoporous PDA (hmPDA) nanoparticles integrated into a transcutaneous microneedle (MN) patch. The hmPDA nanoparticles were synthesized via template-assisted fabrication process, subsequently loaded with RES, and surface-functionalized with a targeting peptide. These multifunctional nanoparticles were then incorporated into a dual-barbed MN array fabricated from a γ-polyglutamic acid (γ-PGA) hydrogel matrix. Upon near-infrared (NIR) laser irradiation, the system induces adipose tissue browning and thermogenesis through synergistic photothermal effects and cargo release.

Statistical analyses in Figure 1 were performed using Origin 2024. Other data were analyzed using GraphPad Prism (version 10.0 for iOS; GraphPad Software, CA, USA). Group comparisons were subjected to Student’s *t*-test (two groups) or one-way analysis of variance (more than two groups). Statistical significance was defined as: ns, not significant, *P < 0.05, **P < 0.01, ***P < 0.001, ****P < 0.0001.

## Results

### Preparation and characterization of RES@hmPDA-Dpep

**Figure 2:**
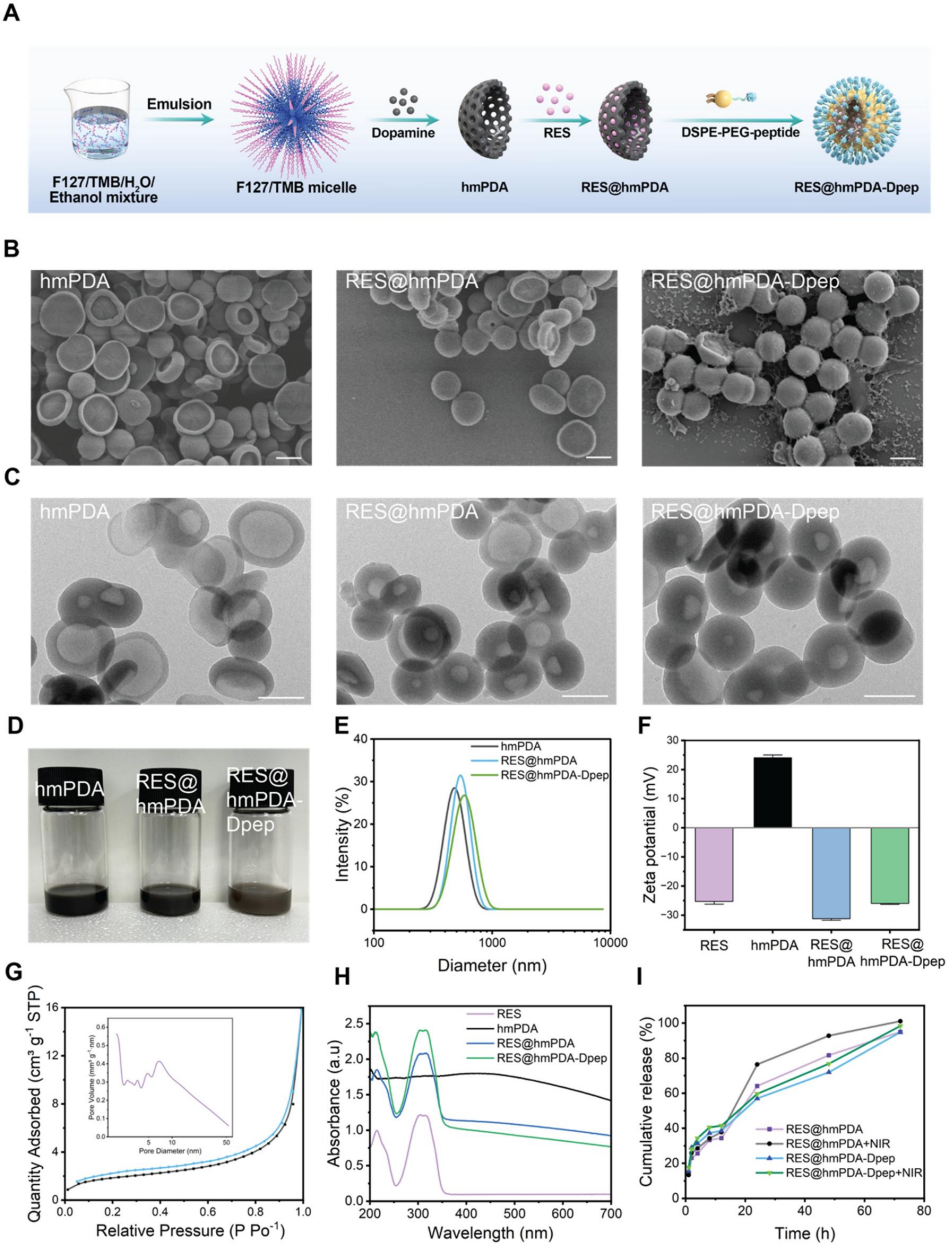
Fabrication and characterization of hmPDA, RES@hmPDA and RES@hmPDA-Dpep. (**A**) Schematic illustration of the stepwise fabrication process of RES@hmPDA-Dpep. (**B**) Representative SEM and (**C**) TEM images of different nanoparticles, scale bar = 500 nm. (**D**) Optical photographs of aqueous dispersions of different groups. (**E**) Diameter distribution of different nanoparticles. (**F**) Zeta potential measurements (*n* = 3) demonstrating surface charge modulation upon sequential functionalization steps. (**G**) Nitrogen adsorption–desorption isotherms and inserted pore size distribution. (**H**) UV–vis spectra (range from 200 to 700 nm) of RES, hmPDA, RES@hmPDA and RES@hmPDA-Dpep. (**I**) Cumulative RES release from RES@hmPDA and RES@hmPDA-Dpep in simulated body fluid (pH 7.3) with or without laser irradiation at different time points.

Hemispherical mesoporous polydopamine nanoparticles were fabricated by a one-step nanoemulsion-guided assembly approach based on our previous work [25]. We first determined that the water-to-ethanol ratio was the decisive factor for the successful emulsification (Supplementary Figure S1). When the 1,3,5-trimethylbenzene (TMB) content increased in the mixture, the resulting PDA shells became more complete and porous, while the particle size of mPDA decreased. A more uniform particle population was obtained when the volumes of ammonia and TMB were set at 0.4 and 1 mL, respectively (Supplementary Figure S2). More bowl-like mPDA emerged upon reducing the dosage of dopamine hydrochloride (Supplementary Figure S3). Ultimately, the homogeneous hemispherical mPDA was isolated via gradient centrifugation. The optimized multi-step process for fabricating the nanoparticles was summarized in Figure 2A. To elucidate the formation mechanism of hmPDA, we provide time-resolved TEM images (0–120 min) collected during synthesis in Supplementary Figure S4, revealing a clear kinetic evolution from ill-defined aggregates at early time points to progressively formed concave intermediates and ultimately well-defined hemispherical particles with a stable concavity. The morphology of hmPDA, RES@hmPDA and RES@hmPDA-Dpep was determined by SEM (Figure 2B). HmPDA and RES@hmPDA exhibited a bowl-like shape, whereas RES@hmPDA-Dpep displayed a more spherical morphology. Based on DLS results, the mean particle size of hmPDA, RES@hmPDA and RES@hmPDA-Dpep was about 485 ± 86, 548 ± 97 and 585 ± 112 nm, respectively (Figure 2E). Generally, modification with DSPE-PEG-CKGGRAKDC enlarged the size of the nanoparticle. TEM images further revealed the inner structures: hmPDA resembled a gauzy disc, whereas RES@hmPDA-Dpep appeared more opaque, indicating increased shell thickness (Figure 2C). The DSPE-PEG-CKGGRAKDC conjugation uniformly coated the entire surface of hmPDA, resulting in a more spherical morphology. Photographic images of nanoparticle dispersions presented a distinct color change after coating with DSPE-PEG-CKGGRAKDC (Figure 2D), with a lighter color observed in RES@hmPDA-Dpep solution. The observed color change likely resulted from the altered surface chemistry and nanoparticle size change after surface modification with DSPE-PEG-CKGGRAKDC. We further evaluated the colloidal stability of mPDA, hmPDA and RES@hmPDA-Dpep in PBS by monitoring time-dependent sedimentation and DLS-measured hydrodynamic size (Supplementary Figure S6). Compared with spherical mPDA, hmPDA showed slower visible sedimentation and improved size stability over time, and DSPE-PEG-CKGGRAKDC functionalization further improved dispersion stability, yielding the most stable suspension among the three formulations.

Zeta potential measurements indicated a change from +25 mV to −30 mV after RES loading, with the final average zeta potential of −26.9 mV (Figure 2F). Brunauer–Emmett–Teller (BET) test confirmed that hmPDA possessed a mesoporous structure (Figure 2G), with the pore size primarily ranging from 5 to 50 nm. The absorption spectrum of RES@hmPDA-Dpep exhibited a distinct peak at 304 nm, which corresponds to the characteristic peak of RES, indicating the successful drug loading (Figure 2H). The LC of RES was calculated to be 96%. The *in vitro* drug release profiles revealed distinct kinetic behaviors among the different formulations (Figure 2I). All groups exhibited a similar initial burst release phase within the first 12 h. However, the RES@hmPDA-Dpep nanoparticles demonstrated a slower release rate than the RES@hmPDA group after 24 h, suggesting that the outer peptide layer further delayed RES release. Notably, NIR laser irradiation facilitated RES release, particularly at 24 and 48 h. Collectively, these results validate the successful synthesis and functional efficacy of RES@hmPDA-Dpep as a controlled-release nanocarrier.

### Characterization and thermal properties of RES@hmPDA-Dpep/MN

**Figure 3:**
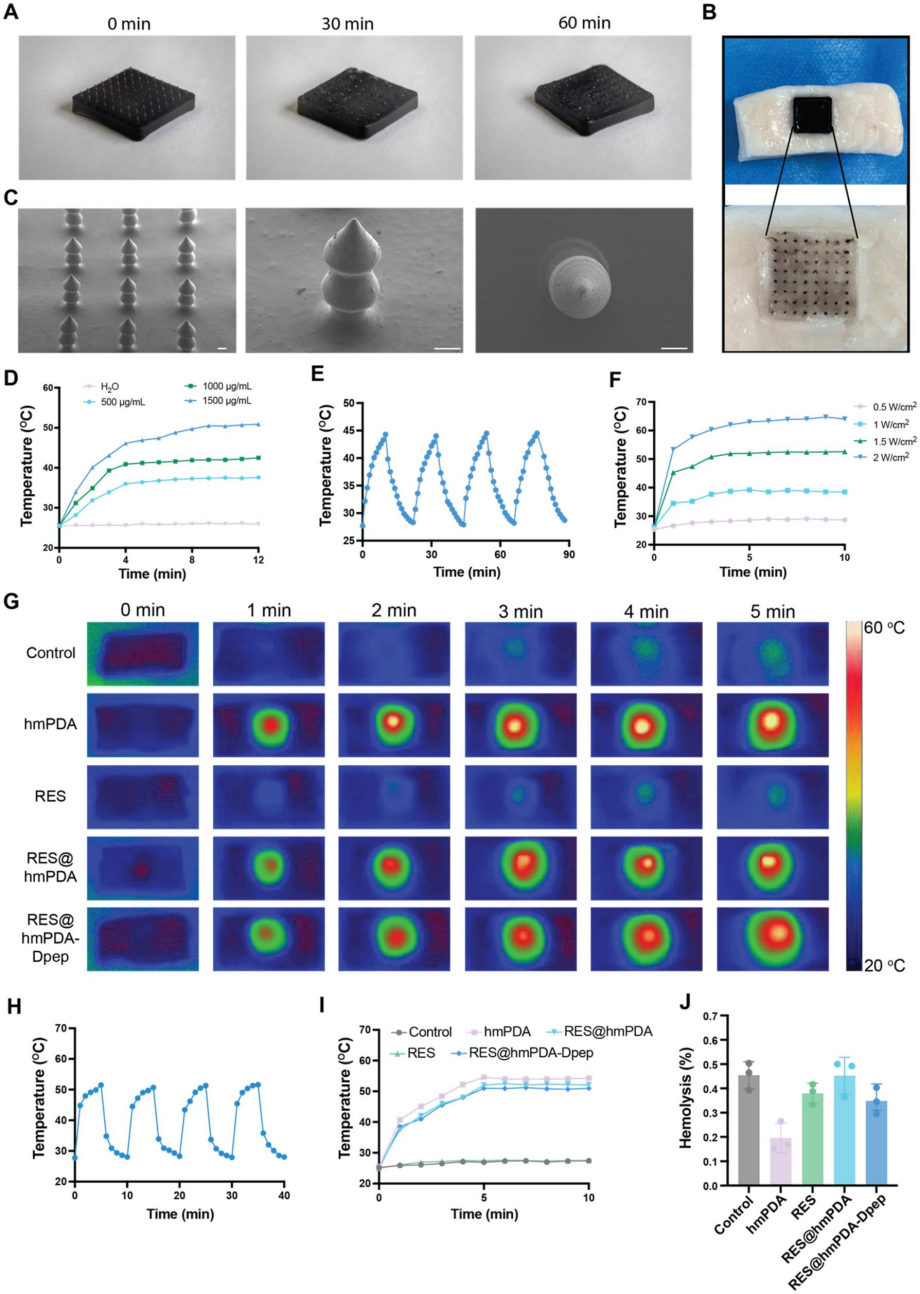
Preparation and photothermal properties of RES@hmPDA-Dpep/MN. (**A**) Sequential optical images of RES@hmPDA-Dpep/MN pulled out from porcine adipose tissue at different time points. (**B**) Light images of penetrated porcine tissue. (**C**) SEM images of RES@hmPDA-Dpep/MN from oblique and vertical view, scale bar = 200 μm. (**D**) Temperature variation curves obtained at various concentrations of RES@hmPDA-Dpep suspension during 20-min irradiation with a laser power density of 1.5 W cm^−2^. (**E**) Temperature variation curves of 1.5-mg mL^−1^ suspensions irradiated at 1.5 W cm^−2^ for four on−off cycles. (**F**) Temperature changes for a 1.5-mg mL^−1^ RES@hmPDA-Dpep suspension at varied power densities over 10 min. (**G**) Thermal images and (**I**) temperature changes of MNs loading different materials in porcine tissue under 1.5-W cm^−2^ irradiation. (**H**) Temperature changes for RES@hmPDA-Dpep/MN irradiated at 1.5 W cm^−2^ for four on−off cycles. (**J**) Quantitative analysis for hemolysis test of different microneedle groups.

The synthesized nanoparticles were mixed with γ-PGA to prepare the pre-gel solution, which was subsequently cast into the patterned molds to fabricate RES@hmPDA-Dpep loaded MN patches. After overnight drying, the patches were collected for characterization. The MNs were sufficiently sharp to penetrate the porcine tissue, and they dissolved gradually within 60 min (Figure 3A), with needle tips remaining embedded in excised adipose tissue (Figure 3B, Supplementary Figure S9). The morphology of hmPDA patches was evaluated by SEM, showing a double barbed shape, with a 900-μm depth for piercing (Figure 3C). Fluorescence imaging of skin cross-sections after MN insertion further confirmed that Rho-labeled hmPDA was successfully delivered into the subcutaneous adipose layer, where the rhodamine signal (red) showed pronounced colocalization with perilipin-positive adipocytes (green) (Supplementary Figure S10A). In addition, mechanical compression testing indicated that the MN patch could withstand an applied force of approximately 8 N (Supplementary Figure S10B).

The photothermal conversion performance of hmPDA, mPDA and RES@hmPDA-Dpep was evaluated under 808-nm NIR laser irradiation. To compare the performance of hemispherical hmPDA and spherical mPDA, we performed a head-to-head evaluation under identical conditions. Under low drug-feeding conditions, hmPDA and mPDA showed high-loading efficiencies similarly; when the RES feeding amount was increased, hmPDA exhibited a clearer advantage in LC, while both morphologies showed comparable photothermal conversion behavior; notably, both formulations reached a similar maximal cumulative release (∼90% at 72 h), with hmPDA displaying a faster early-phase release profile (Supplementary Figure S5). For RES@hmPDA-Dpep, at a concentration of 1500 μg mL^−1^, the temperature of the suspension was raised from 25.5 to ∼46°C within 5 min when exposed to 1.5 W cm^−2^ laser irradiation, demonstrating a concentration-dependent heating effect (Figure 3D). Similarly, the suspension also exhibited a laser power-dependent heating response (from 0.5 to 2 W cm^−2^) (Figure 3F). Moreover, no obvious variation was observed in temperature during four on–off thermal cycles, indicating remarkable stability and recyclability of the RES@hmPDA-Dpep (Figure 3E).

Likewise, the photothermal conversion capacity of RES@hmPDA-Dpep/MN was further assessed on excised porcine subcutaneous adipose tissue. No significant difference was found in the magnitude of temperature rise after 10-min irradiation among the hmPDA, RES@hmPDA and RES@hmPDA-Dpep groups (Figure 3G and I) under a 1.5 W cm^−2^ laser power, while only a slight increase in temperature was observed in the empty MN and RES groups. Simultaneously, RES@hmPDA-Dpep/MN showed stable and repeatable thermal performance, as evidenced by the time-temperature plot (Figure 3H). Notably, RES@hmPDA-Dpep/MN patch exhibited a faster temperature increase from 25.0 to 51°C over 5 min, showing greater fluctuation compared to its suspension counterpart. The enhanced light-to-heat conversion efficiency presented by RES@hmPDA-Dpep/MN may be attributed to the drying process in the procedure of MN.

Biocompatibility is crucial for ensuring safe and effective application; therefore, the biosafety of RES@hmPDA-Dpep/MN was investigated using both live/dead staining and hemolysis test. The results revealed no significant toxicity to 3T3-L1 cells after 24 h of incubation with the MN leachate (Supplementary Figure S8B), and the hemolysis percentages remained below 0.5% across all tested groups (Figure 3J, Supplementary Figure S11). These *in vitro* findings suggest that the RES@hmPDA-Dpep/MN possesses favorable thermal properties and biocompatibility, laying a solid foundation for bioapplication. Overall, the patch showed robust photothermal performance and satisfactory biocompatibility, supporting further applications.

### Cellular uptake and inhibition of lipogenesis *in vitro*

**Figure 4:**
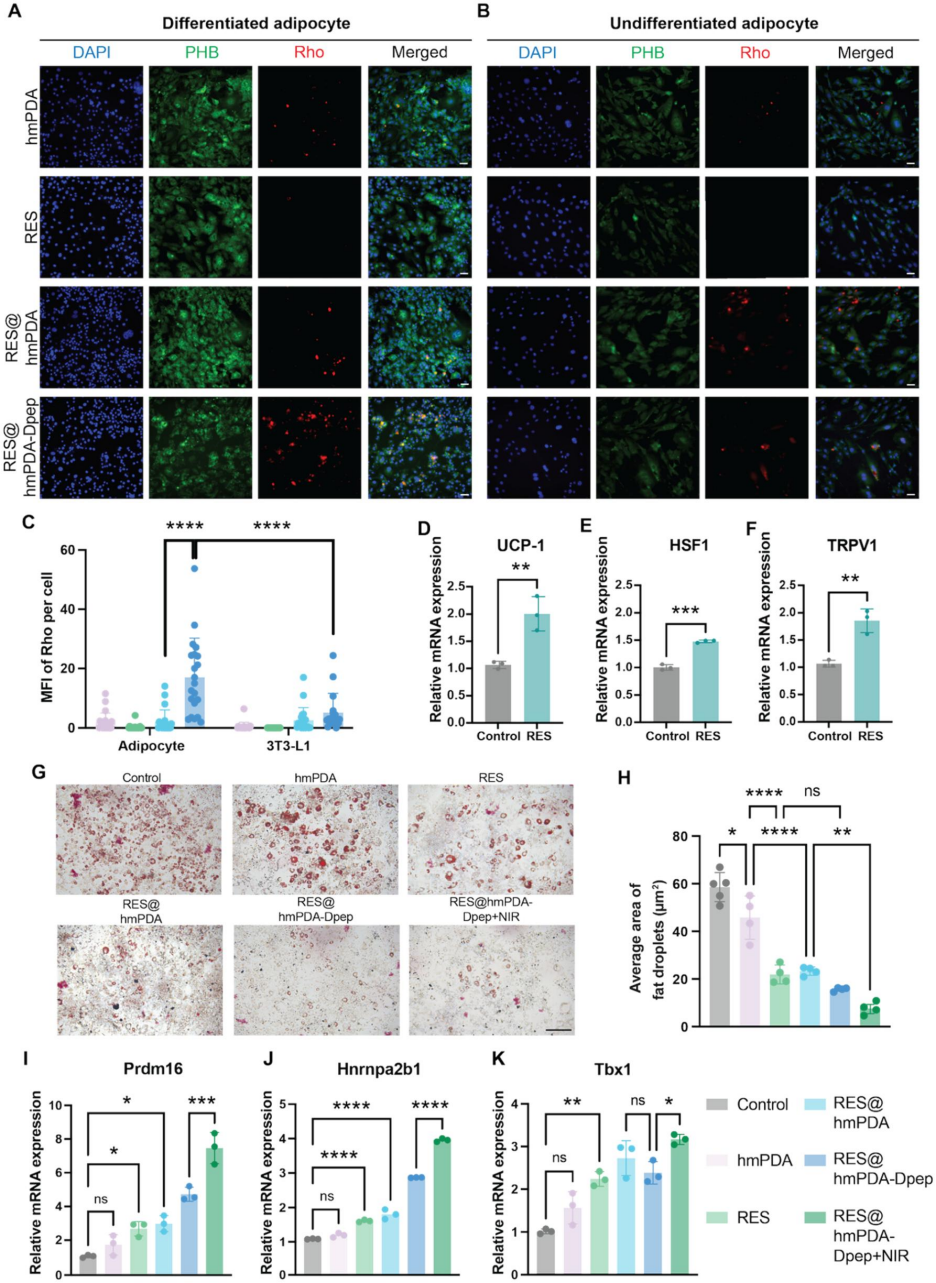
Cellular uptake and *in vitro* lipid-reduction efficacy of RES@hmPDA-Dpep. Representative fluorescence microscopy images of differentiated adipocytes (**A**) and undifferentiated adipocytes (**B**) after incubation with FITC-labeled PHB and rhodamine B (rho)-labeled nanoparticles; nuclei were stained with DAPI, scale bar = 100 μm. (**C**) Quantified mean fluorescence intensity (MFI) of nanoparticles. Relative mRNA expression of (**D**) UCP-1, (**E**) HSF1 and (**F**) TRPV1 in 3T3-L1 cells cultured in 41°C (*n* = 3). (**G**) Oil-red-O staining of intracellular lipid droplets in mature adipocytes and (**H**) quantified fat droplets area, scale bar = 200 μm. Relative mRNA expression of (**I**) Prdm16, (**J**) Hnrnpa2b1 and (**K**) Tbx1 in mature adipocytes treated with different groups. *P < 0.05, **P < 0.01, ***P < 0.001, ****P < 0.0001.

To determine a safe and effective concentration of RES, we set up a concentration gradient and assessed its impact on cell viability by CCK-8 assay (Supplementary Figure S8A). Finally, the 25-μM RES was selected for subsequent experiments. Meanwhile, we evaluated the dose-dependent cytocompatibility of hmPDA nanoparticles by CCK-8 assay (Supplementary Figure S7). After 48-h incubation, 3T3-L1 cell viability remained high across the tested concentration range, supporting the favorable cytocompatibility of hmPDA. To verify whether the targeting peptide could enhance the uptake efficiency of combined nanoparticles by adipocytes, co-immunofluorescence staining of PHB (green) and RES@hmPDA-Dpep nanoparticles (red) was conducted. The results suggested that expression of PHB is higher in mature adipocytes than in premature adipocytes (Figure 4A and B). Moreover, green fluorescence co-localized with red fluorescence was markedly stronger in RES@hmPDA-Dpep groups for both cell types (Figure 4C), visually confirming that peptide CKGGRAKDC indeed boosts nanoparticle uptake by adipocytes. To further evaluate tissue-level distribution, epi-fluorescence imaging was performed using an in *vivo* imaging system (IVIS) (Supplementary Figure S12). A strong fluorescence signal was detected in the inguinal adipose tissue at 24 h after MN insertion, and a weak signal was also observed in visceral adipose tissue at 48 h. No detectable fluorescence was found in other major organs under the same imaging settings, suggesting preferential accumulation in adipose tissues and supporting the targeting performance of the formulation.

Previous studies have revealed that RES can upregulate the expression of HSF1, which is indispensable for the effects of hyperthermia against obesity in mice [21, 22]. To verify our hypothesis that RES could potentiate mild hyperthermia-induced thermogenesis, we tested the change in thermogenetic gene transcripts expression. When cultured at 41°C environmental temperature, adipocytes treated with RES exhibited significantly higher expression of UCP-1 than the control group (37°C) (Figure 4D). UCP-1 is a key marker for thermogenesis and browning of white adipocytes [26]. Furthermore, transient receptor potential vanilloid 1 (TRPV1), a classic heat-responsive protein, can be activated at 41–43°C, takes responsibility in orchestrating cellular response to heat [27]. Together with HSF1, these proteins also engaged in metabolic health and obesity [28]. High temperatures induced elevated expression of UCP-1 and TRPV1; the effect was enhanced by RES supplementation (Figure 4E and F). After confirming synergistic effect of RES and hyperthermia, the lipid-reduction capacity of RES@hmPDA-Dpep was evaluated by Oil-Red-O staining *in vitro*. Mature adipocytes exhibited large unilocular lipid droplets, which were fragmented into a multilocular appearance by the introduction of RES, with further lipid reduction observed in the RES@hmPDA-Dpep+NIR group (Figure 4G and H). No difference was observed between RES@hmPDA and RES@hmPDA-Dpep groups, suggesting that targeting advantage is not evident, likely due to the simplicity of the single-cell *in vitro* model, which failed to mimic the complex environment *in vivo*. Next, RT-qPCR was carried out to investigate the influence on brown fat genes after receiving PBT.

PR domain-containing 16 (Prdm16) is required for the “browning” of white fat and the regulation of the thermogenic program in beige adipocytes [29]. Tbx1, compared with previously identified marker Prdm16, is a newly established beige adipose cell biomarker, which can distinguish beige adipocytes from other adipocyte types [30]. We also studied Hnrnpa2b1, a downstream target of HSF1 to stabilize the transcripts of metabolic genes. Functionally, all these beige-selective and thermoregulatory genes were upregulated in RES@hmPDA-Dpep + NIR group (Figure 4I–K).

Collectively, these findings indicated that RES enhanced the ability of induced beige adipocytes to sense the temperature rise and respond with hyperthermia-induced thermogenesis. In addition, the RES@hmPDA-Dpep nanoparticle with laser irradiation effectively reduced lipogenesis *in vitro*.

### Anti-obesity performance in diet-induced obese mice

**Figure 5:**
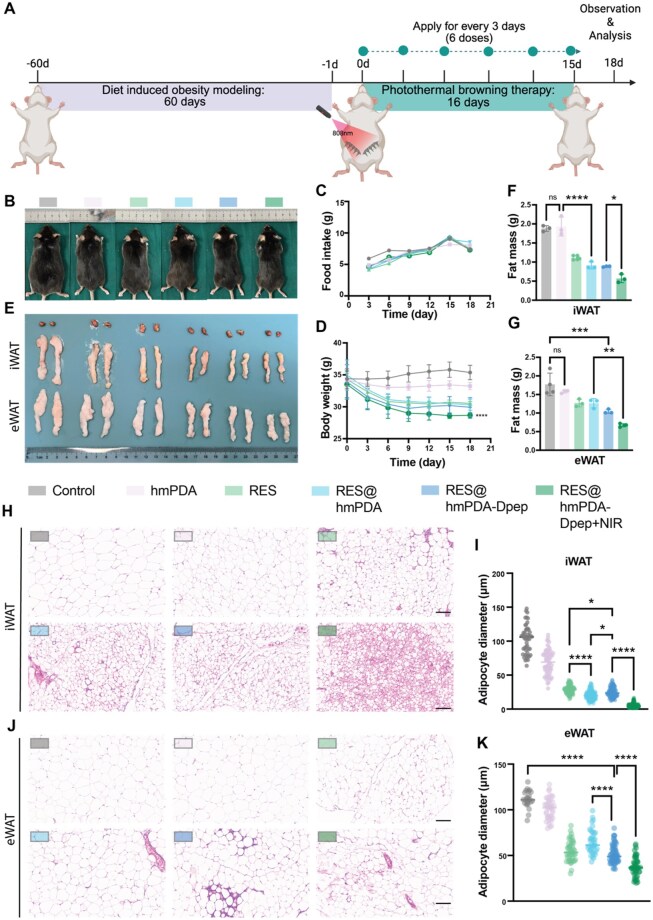
*In vivo* anti-obesity efficacy evaluation of RES@hmPDA-Dpep/MN therapy. (**A**) Schematic diagram illustrating the *in vivo* experimental setup. (**B**) Representative images of mice and (**E**) excised BAT, eWAT and iWAT from different groups on day 18 are shown. Physiological monitoring of (**C**) food intake and (**D**) body weight over time. The quantified fat mass of (**F**) iWAT and (**G**) eWAT. H&E-stained sections of (**H**) iWAT and (**J**) eWAT (scale bar = 400 μm), and (**I**, **K**) the average adipocyte sizes calculated by images. *P < 0.05, **P < 0.01, ***P < 0.001, ****P < 0.0001.

Next, we sought to test the anti-obesity effects of our PBT *in vivo*. C57BL/6 mice were first fed a high-fat diet for 2 months to establish the diet-induced obese mice model, then the different MN patches were applied to the bilateral inguinal region once every 3 days, lasting for 18 days (Figure 5A). Mice in the RES@hmPDA-Dpep/MN+NIR group additionally received NIR laser (808 nm), with the temperature maintained within the range of 41–43°C with a thermal imager (Supplementary Figure S13). With similar initial body weights at the beginning and continuous high-fat (60%) feeding during the therapy, mice in the control group gained 4.33 ± 1.95% (from 35.6 ± 2.4–37.1 ± 1.9 g) of body weight, whereas mice in the RES/MN, RES@hmPDA/MN and RES@hmPDA-Dpep/MN groups lost 12.2 ± 3.7%, 14.7 ± 1.4% and 15.3 ± 1.5% of their body weight, respectively (Figure 5D). The RES@hmPDA-Dpep/MN+NIR group exhibited the greatest weight loss among all groups (16.1 ± 2.5%). Meanwhile, food intake was also monitored throughout the treatment period and presented no significant differences among groups (Figure 5C). At the end of PBT therapy, mice in RES@hmPDA/MN, RES@hmPDA-Dpep/MN and RES@hmPDA-Dpep/MN+NIR groups presented a macroscopical leaner physique (Figure 5B). Subsequently, adipose depots from the interscapular region (brown adipose tissue), the inguinal region (iWAT) and the epididymal area (eWAT) were isolated and examined. Generally, inguinal adipose tissue represents a subcutaneous fat pool, whereas epididymal adipose tissue is considered visceral fat, both of which are regarded as WAT. The WAT depots shrank both in size and mass, corresponding with the observed change of body shape (Figure 5E).

The results of H&E staining further supported the anti-obesity effect observed above. Adipocyte diameters in subcutaneous WAT were significantly reduced, and adipocytes displayed a more “brown-like” morphology in RES@hmPDA-Dpep/MN+NIR group (Figure 5H and I). Analogously, the size of adipocytes in visceral WAT declined to some extent, although morphological changes were less pronounced compared to iWAT (Figure 5J and K). Collectively, the *in vivo* anti-obesity effect of the PBT was demonstrated by the reduced excess body fat and promoted beige adipocyte formation.

### RES@hmPDA-Dpep/MNs reduced body fat and ameliorated systemic metabolism

**Figure 6:**
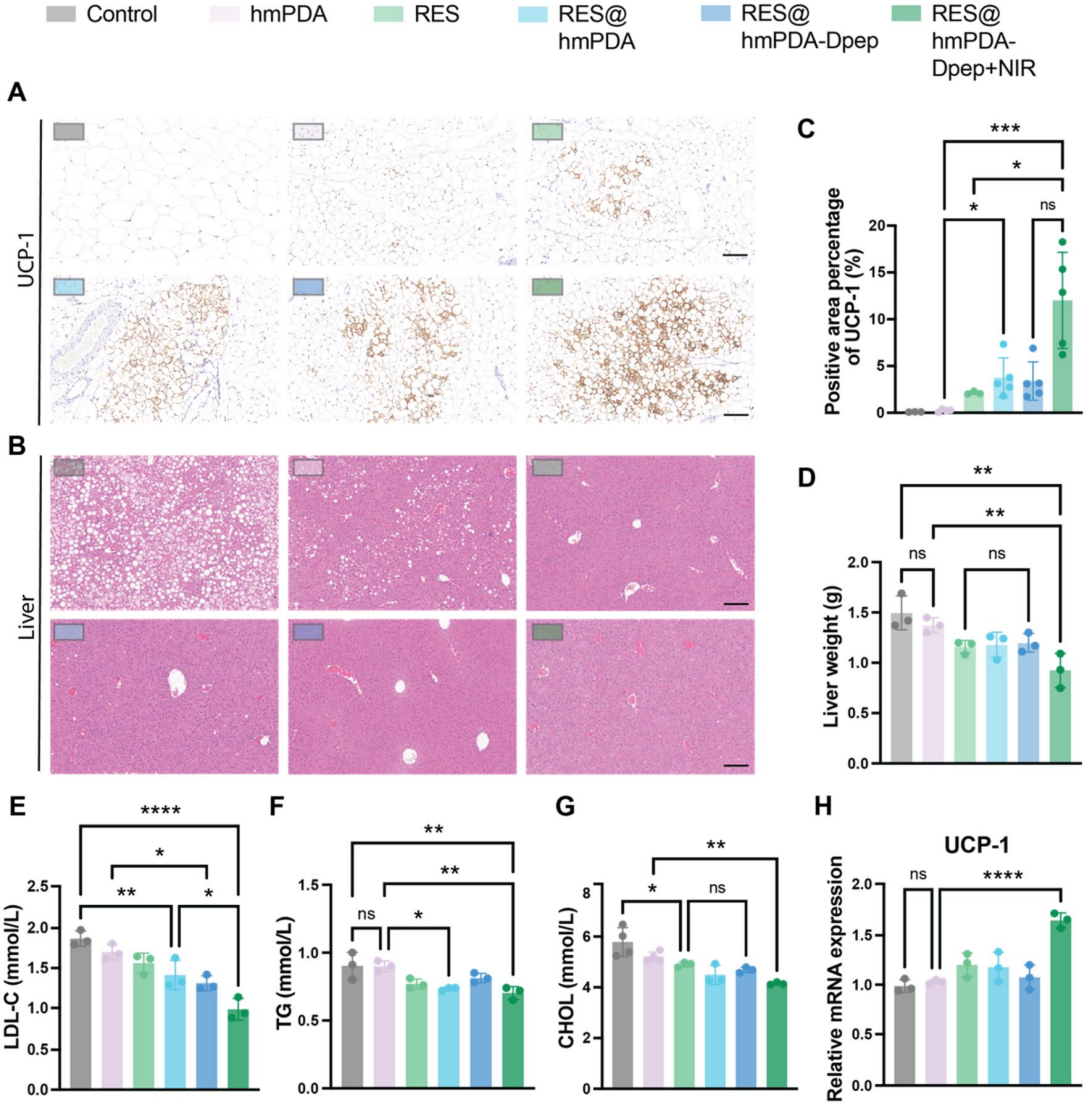
*In Vivo* metabolic reprogramming and lipid profile modulation by RES@hmPDA-Dpep/MN therapy. (**A**) The thermogenesis effect was determined by immunohistochemical staining of UCP-1, and the (**C**) quantified positive expression area in iWAT (*n* = 5) (scale bar = 400 μm). (**B**) The severity of fatty liver was observed by H&E staining images of livers and (**D**) their weight. The serum lipid profiles of (**E**) LDL-C, (**F**) TG and (**G**) cholesterol were examined by a biomedical analyzer. (**H**) The mRNA expression level of UCP-1 in iWAT (*n* = 3). *P < 0.05, **P < 0.01, ***P < 0.001, ****P < 0.0001.

In mice, brown adipocytes (lie in brown adipose tissue) and beige adipocytes (also called inducible brown adipocytes) mediate the adaptive non-shivering thermogenic response and also take metabolic roles beyond thermogenesis. To investigate the physiological impacts of PBT more comprehensively, we conducted a more detailed investigation. A remarkable improvement in UCP-1 expression of subcutaneous adipose tissue (Figure 6A and C) was observed in immunohistochemical stains as well as in qPCR results (Figure 6H), indicating that iWAT underwent a “shift” from white-to-brown-like adipose tissue, which is highly consistent with the *in vivo* observations. Since UCP1 is a key mediator of mitochondrial heat production, this suggests that PBT promotes thermogenesis and enhances energy expenditure *in vivo*.

Obesity is often accompanied by systemic metabolic dysfunction; for example, the circulation of lipids secreted from excess WAT contributes to hepatic lipid accumulation, leading to nonalcoholic fatty liver disease. To evaluate the effects of PBT on lipid metabolism, the degree of lipid droplets in the liver was assessed, and levels of serum lipids were measured. Histological examination of liver sections showed widespread lipid droplet accumulation in the control group, whereas RES-treated groups exhibited reduced hepatic lipid accumulation and decreased liver weight (Figure 6D). No significant differences were found between RES-treated groups (Figure 6B). Circulating LDL-C, TGs and cholesterol were analyzed, and all those parameters decreased after PBT (Figure 6E–G), indicating the amelioration of systemic dyslipidemia.

In conclusion, these results confirm that PBT effectively reduces body fat, attenuates hepatic steatosis, and improves dyslipidemia, highlighting its potential as a therapeutic approach for obesity-related metabolic dysregulation.

## Discussion

The integrated RES@hmPDA-Dpep/MN possessed a notable fat-loss effect and the ability to mitigate the obesity-associated metabolic disturbances. These outcomes can be attributed to the unique structural and functional features of the hemispherical mesoporous polydopamine (hmPDA) nanoparticles developed in this study. Although classic routes of synthesizing spherical mesoporous polydopamine (mPDA) nanoparticles have been extensively studied for their photothermal properties and drug delivery capabilities since their initial development, ongoing efforts aim to optimize synthesis strategies and identify novel morphologies that offer enhanced functionalities or broader applications [31]. Previous studies have stumbled across a synthesis route of hemispherical mPDA [32, 33], and recently Zheng *et al.* [34] also manufactured hmPDA using a one-step method; however, our study presents a more detailed synthesis strategy, yielding well-defined bowl-like nanoparticles that retained thermal properties of classic spherical mPDA. Intriguingly, the average diameter of hmPDA (350–550 nm) was significantly larger than that of typical spherical mPDA (usually 200–300 nm), suggesting differences in the self-assembly dynamics during the process [35, 36]. However, the precise formation mechanism remains unclear and merits further mechanistic investigation.

To enhance stability and functionality, we uniformly coated the entire surface of the hmPDA nanoparticles with DSPE-PEG, an amphiphilic copolymer featuring a hydrophobic lipid core (DSPE) and a hydrophilic PEG shell [37]. The core-shell structure enables DSPE-PEG2000 to self-assemble into spherical micelles with a 12-nm hydrodynamic size, but their limited size and fixed conformation constrain drug LC and colloidal stability in physiological environments [38]. Anchoring DSPE-PEG onto hmPDA combines the advantages of both components: the inside hmPDA retained the photothermal and drug-loading capability of mPDA, while the outside enabled targeted delivery through conjugation with the adipose-homing peptide CKGGRAKDC. Our results confirmed that this targeting peptide significantly improved the uptake efficiency of nanoparticles by mature adipocytes, thereby enhancing therapeutic precision.

The therapeutic efficacy of the RES@hmPDA-Dpep/MN patch was further augmented by the synergistic interaction between RES and mild hyperthermia. RES pretreatment sensitized adipocytes to mild hyperthermia, as evidenced by elevated expression levels of TRPV1 and HSF1, leading to upregulation of UCP1 and promotion of white adipose browning. Such HSF1 activation has not been widely reported for other commonly used browning agents, such as rosiglitazone [39]. A growing body of evidence indicates that many polyphenols can promote WAT browning and increase UCP1 expression; for instance, quercetin and citrus-derived polyphenols such as eriocitrin have been reported to enhance thermogenic gene programs [40]. However, these effects are most commonly attributed to canonical metabolic pathways (e.g. the AMPK/SIRT1/PPAR axis) rather than explicit heat-sensing circuits. Beyond the obesity/browning field, thermosensory and stress-responsive pathways can also be modulated by naturally derived bioactives: TRPV1 is directly activated by capsaicin with well-defined mechanistic and structural evidence, and EGCG auto-oxidation products have been reported to activate TRPV1/TRPA1 in sensory systems [41, 42]. Likewise, polyphenols can engage heat-shock signaling, as RES has been reported to activate HSF1 via a SIRT1-dependent mechanism and curcumin can enhance HSF1 phosphorylation [43]. However, studies that mechanistically integrate the full HSF1/TRPV1–UCP1 cascade in the context of mild hyperthermia–assisted adipose browning remain limited; thus, our work helps address this gap and suggests that additional polyphenols may also converge on this pathway, which warrants future systematic validation. Furthermore, incorporation into hmPDA significantly improved the aqueous solubility and loading efficiency of RES, thereby enhancing its bioavailability and therapeutic potential.

MN-based transdermal delivery systems have attracted considerable attention in recent years due to their minimally invasive nature and high patient compliance [44, 45]. Currently, much interest has been focused on anti-obesity MNs, which address limitations of oral administration, such as first-pass metabolism, and enable localized, controlled drug delivery [46–52]. In line with previous studies, our platform has achieved a notable fat-loss effect and improved systemic metabolic parameters, highlighting its translational potential for clinical obesity interventions.

Although general obesity involves both expansion of subcutaneous and visceral adipose tissue, visceral adiposity is more closely related to metabolic diseases such as insulin resistance, type 2 diabetes, atherogenic dyslipidemia and cardiovascular disease [53]. In our study, both iWAT and eWAT were decreased after receiving PBT; however, the response of eWAT was less pronounced than that of iWAT. This discrepancy may be attributed to the superficial delivery route of the MN patch. Although a portion of nanoparticles may enter systemic circulation and affect visceral fat, the majority remain confined to the subcutaneous layer. Thus, future investigations should focus on developing strategies to enhance delivery to deeper adipose depots, broadening the applicability of PBT for comprehensive obesity management. In addition, our experiments lack long-term follow-up and rebound evaluation after drug withdrawal; this will be an important direction for future studies.

## Conclusion

In summary, a targetable, biocompatible and anti-obesity RES@hmPDA-Dpep nanoparticle with a previously unappreciated hemispherical structure of mPDA was successfully constructed. The RES@hmPDA-Dpep/MN+NIR treatment not only reduced body weight without affecting food intake but also ameliorated systemic hyperlipidemia. These therapeutic effects highlight a potential approach to address the clinical need for managing the high prevalence of obesity-related syndromes. Hence, the RES@hmPDA-Dpep/MN+NIR treatment proposed in this article helps address the limited synergy between conventional browning agents and photothermal transducers, and warrants further investigation toward translational applications.

## Supplementary Material

rbag052_Supplementary_Data

## Data Availability

Data will be made available on request.
