# Supplementary material for: NIR-activated adipose-targeted nanocarrier drives local browning and metabolic restoration
Source: Regen Biomater. 2026 Mar 9;13:rbag052. doi: 10.1093/rb/rbag052 (PMC13157220; doi:10.1093/rb/rbag052)
Supplement: rbag052_Supplementary_Data [file rbag052_supplementary_data.zip › Supplementary Data 0227 final.docx]

Supporting Information

**NIR-Activated Adipose-Targeted Nanocarrier Drives Local Browning and Metabolic Restoration**

Ying Li^1#^, Enze Liu^1#^, Pei Wang^1^, Jiayi Liu ^2^, Weijie Jin^3^, Zhen Gao^1*^, Jiayingzi Wu^4*^, Xiansong Wang^1*^

^1^Department of Plastic and Reconstructive Surgery, Shanghai Key Laboratory of Tissue Engineering, Shanghai Ninth People’s Hospital, Shanghai Jiao Tong University School of Medicine, Shanghai 200011, China.

^2^Shenghua Zizhu Academy, Shanghai 200241, China.

^3^Division of Infection and Immunity, Faculty of Medical Science, University College London, London, United Kingdom

^4^Guangdong Key Laboratory for Biomedical Measurements and Ultrasound Imaging, School of Biomedical Engineering, Medical School, Shenzhen University, Shenzhen, 518055, China

^#^These authors contributed equally to this work.

^*^Corresponding author


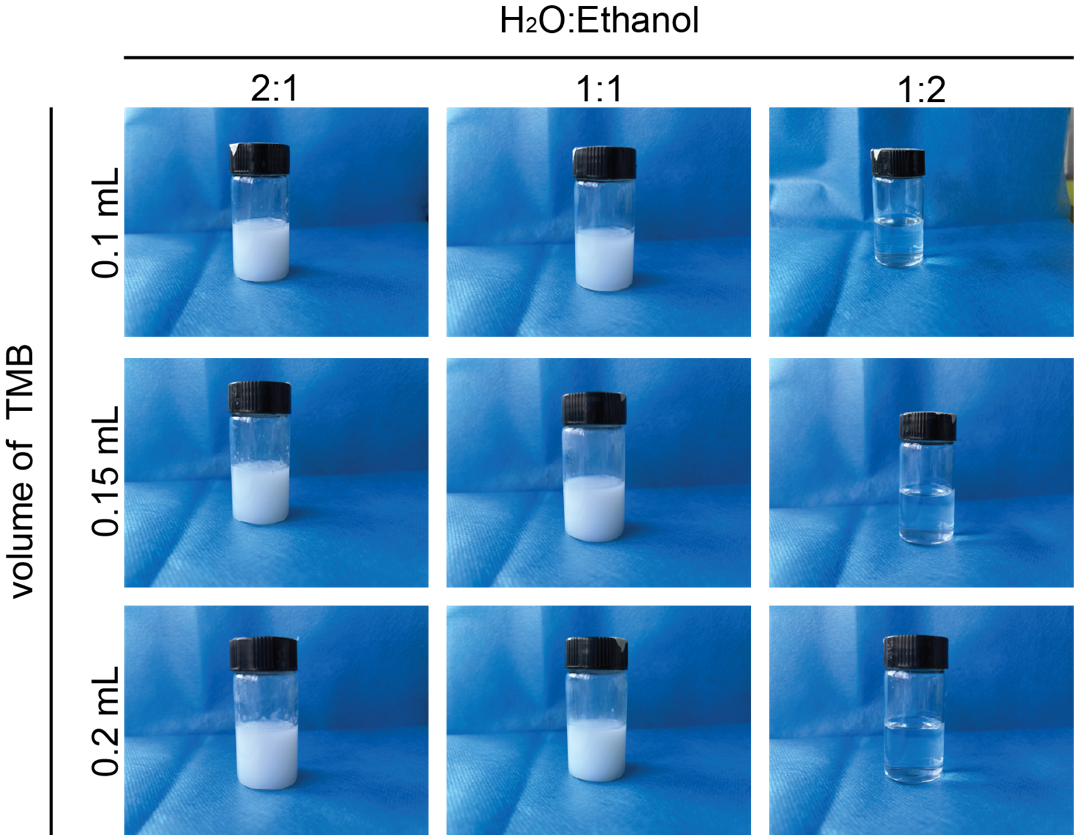


**Figure S1.** Digital photographs of the reaction solution containing F127 and TMB under different proportions of water and ethanol. In a system where H_2_O: ethanol is 1:2, the final solution failed to reach emulsion regardless of the volume of TMB.


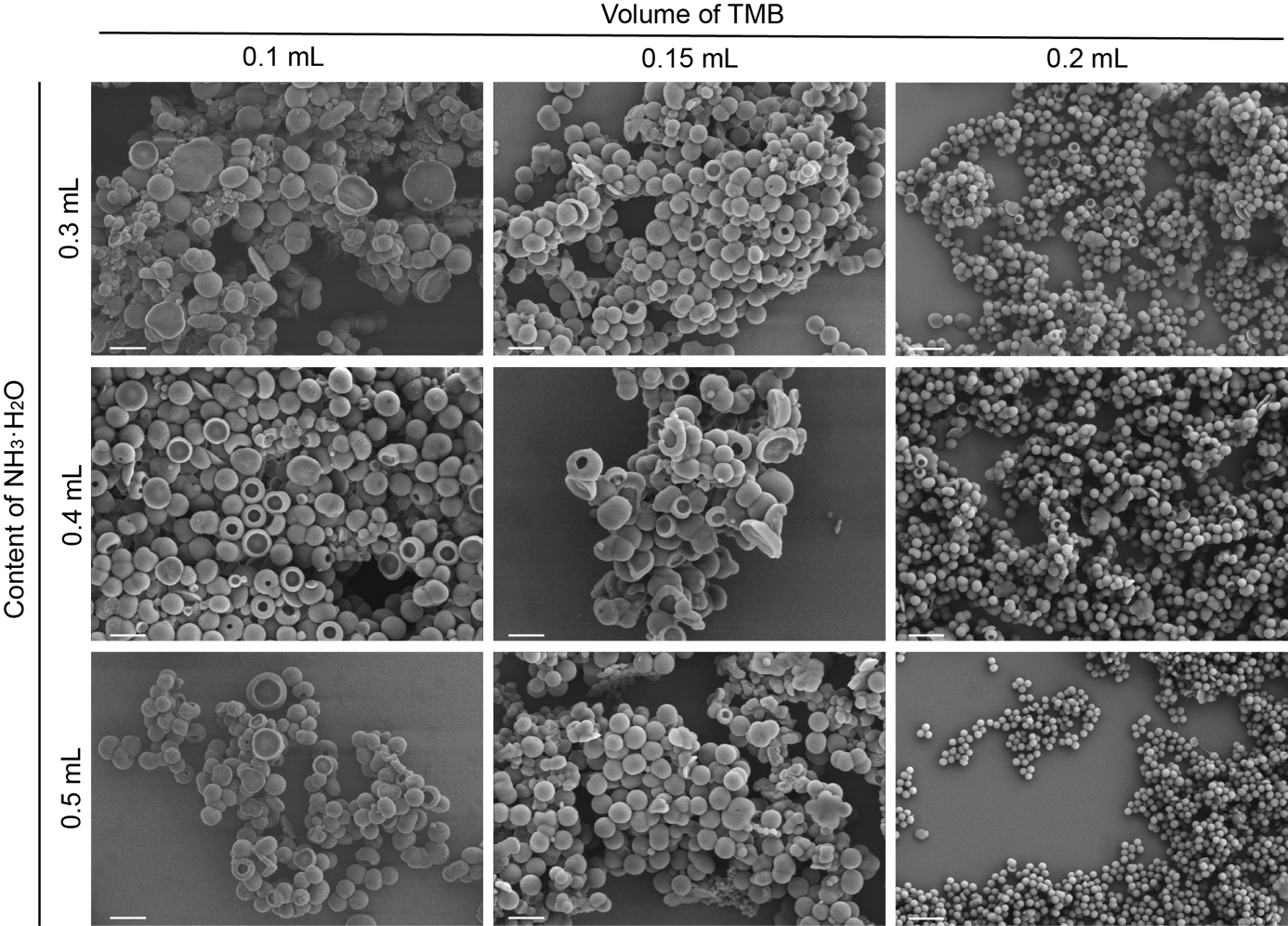


**Figure S2.** TEM images of the mPDA products obtained by changing the content of ammonia and TMB. Scale bar = 1 μm.


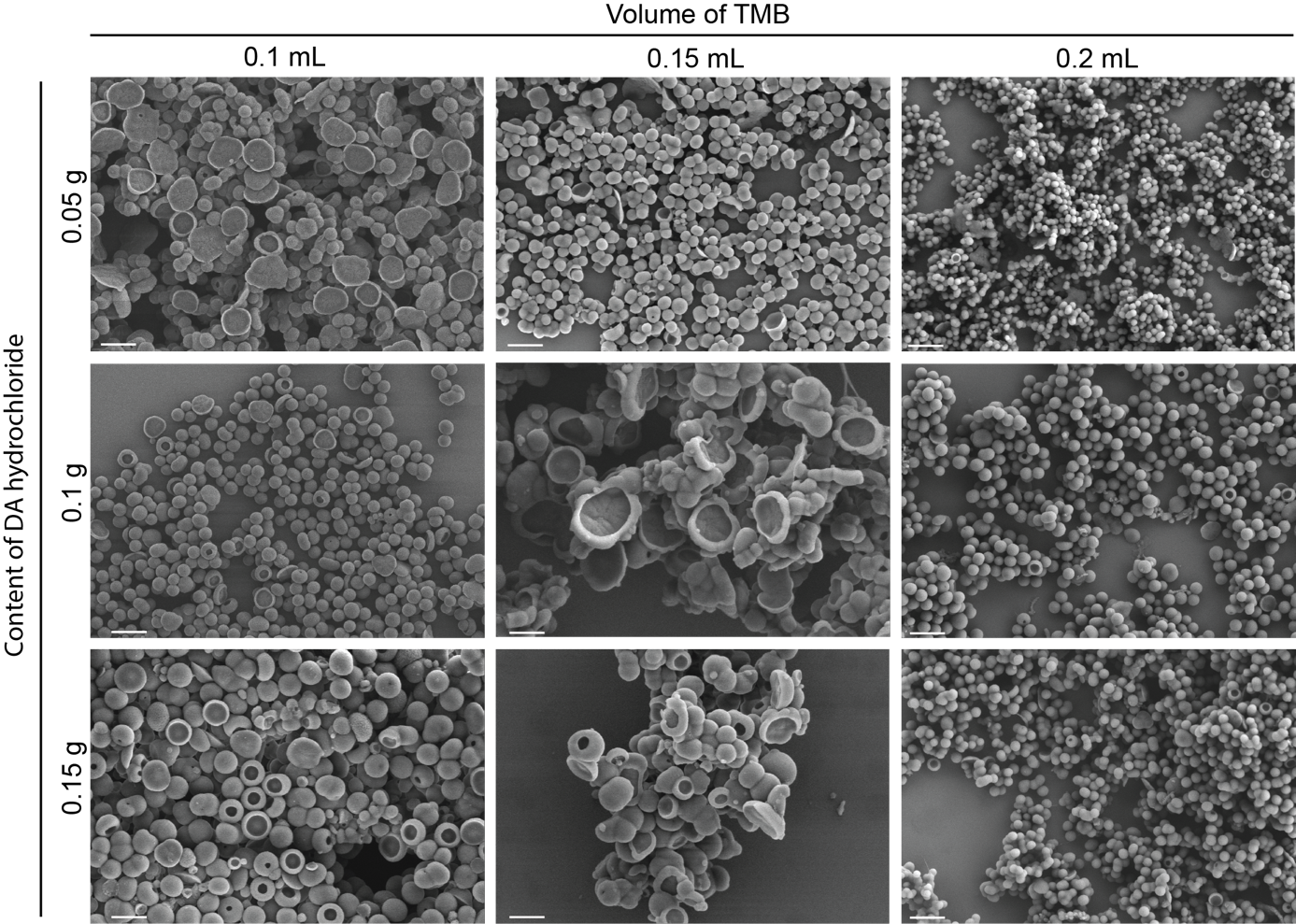


**Figure S3.** TEM images of the mPDA products obtained by changing the content of DA hydrochloride and TMB. Scale bar = 1 μm.


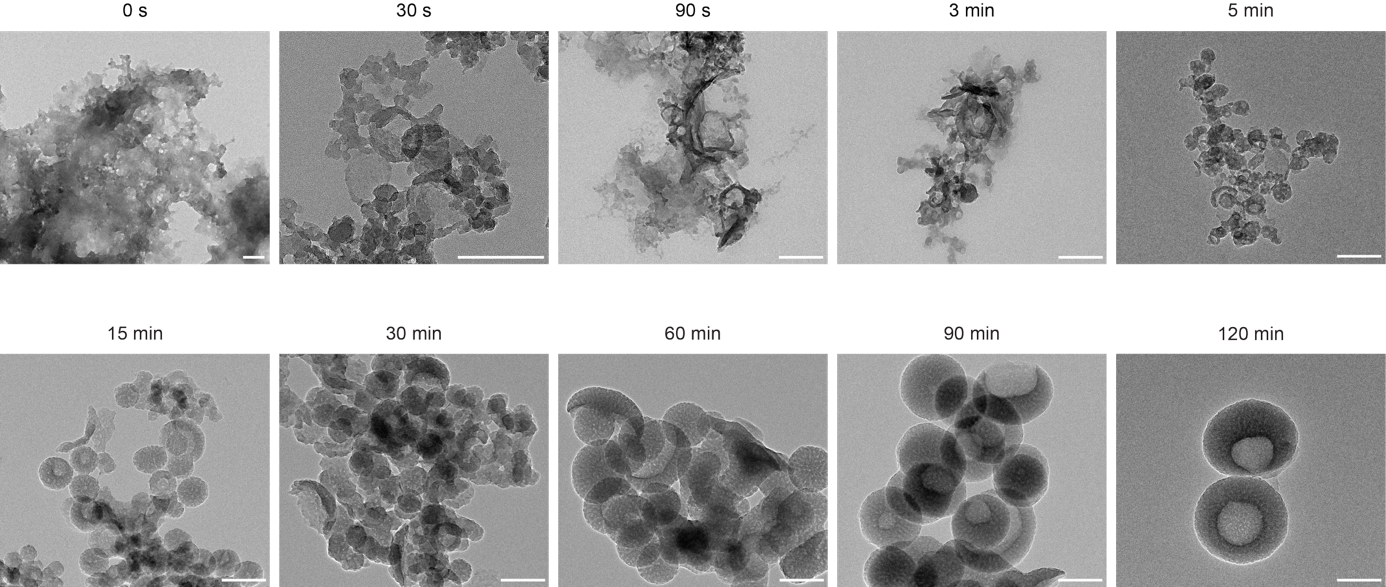


**Figure S4.** TEM images of the hmPDA products at different time points. Scale bar = 200 nm.


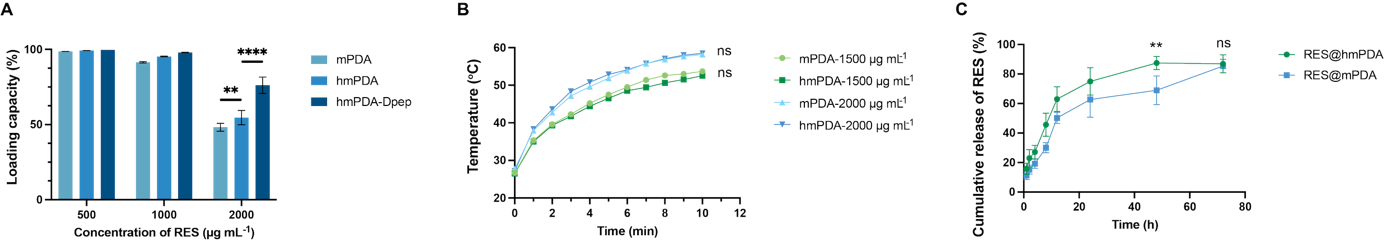


**Figure S5.** Comparison between hmPDA and mPDA particles in drug loading capacity, photothermal conversion and drug release curve. ** p<0.01, **** p< 0.0001


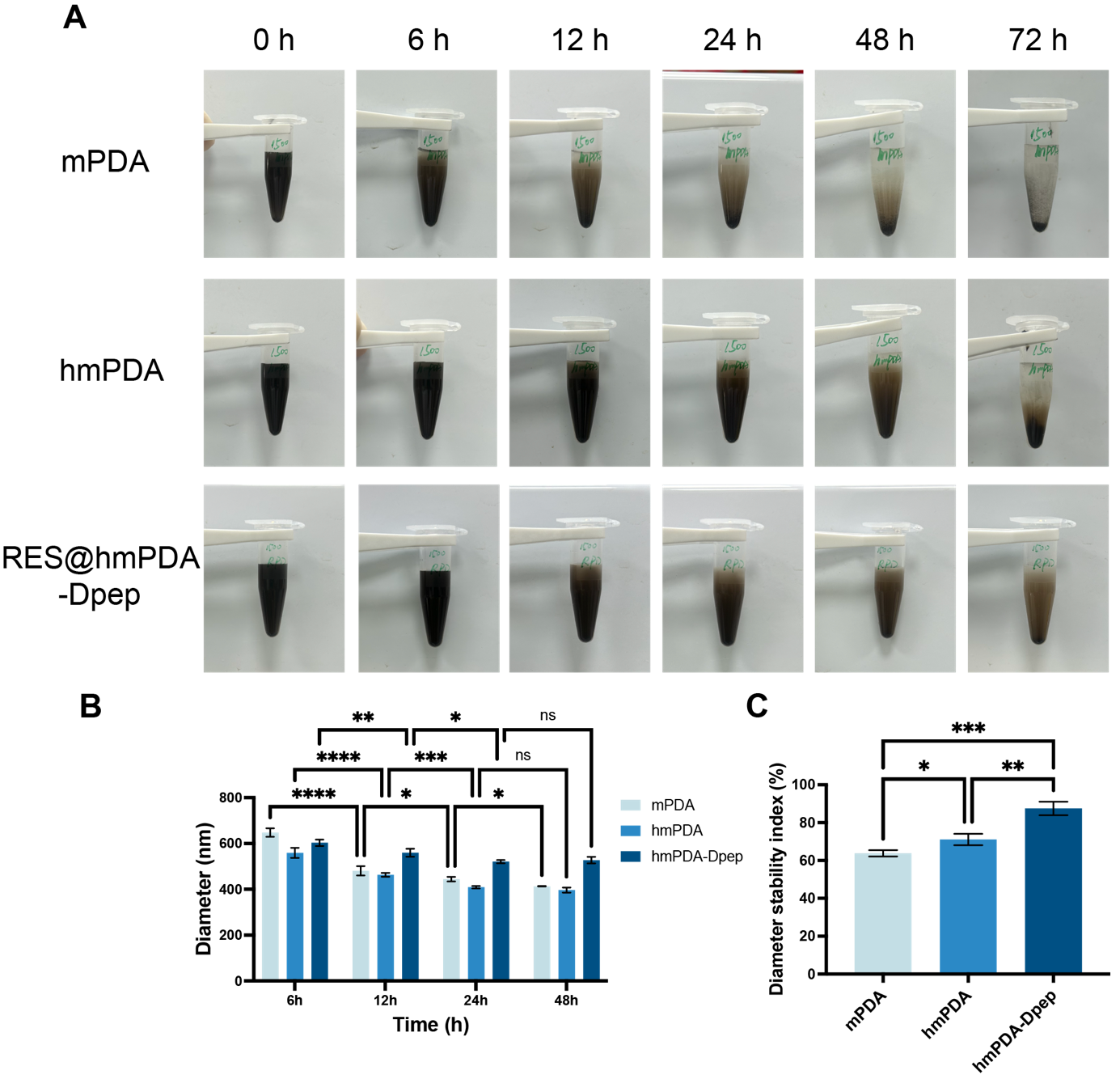


**Figure S6. Colloidal stability of nanoparticles in PBS.** (**A**) Time-dependent sedimentation behavior of different nanoparticles in PBS. (**B**) Hydrodynamic diameter measured by DLS at different time points (mean ± SD, n = 3). (**C**) Diameter stability index expressed as the size ratio (D 48 h/ D 6 h) (mean ± SD, n = 3).


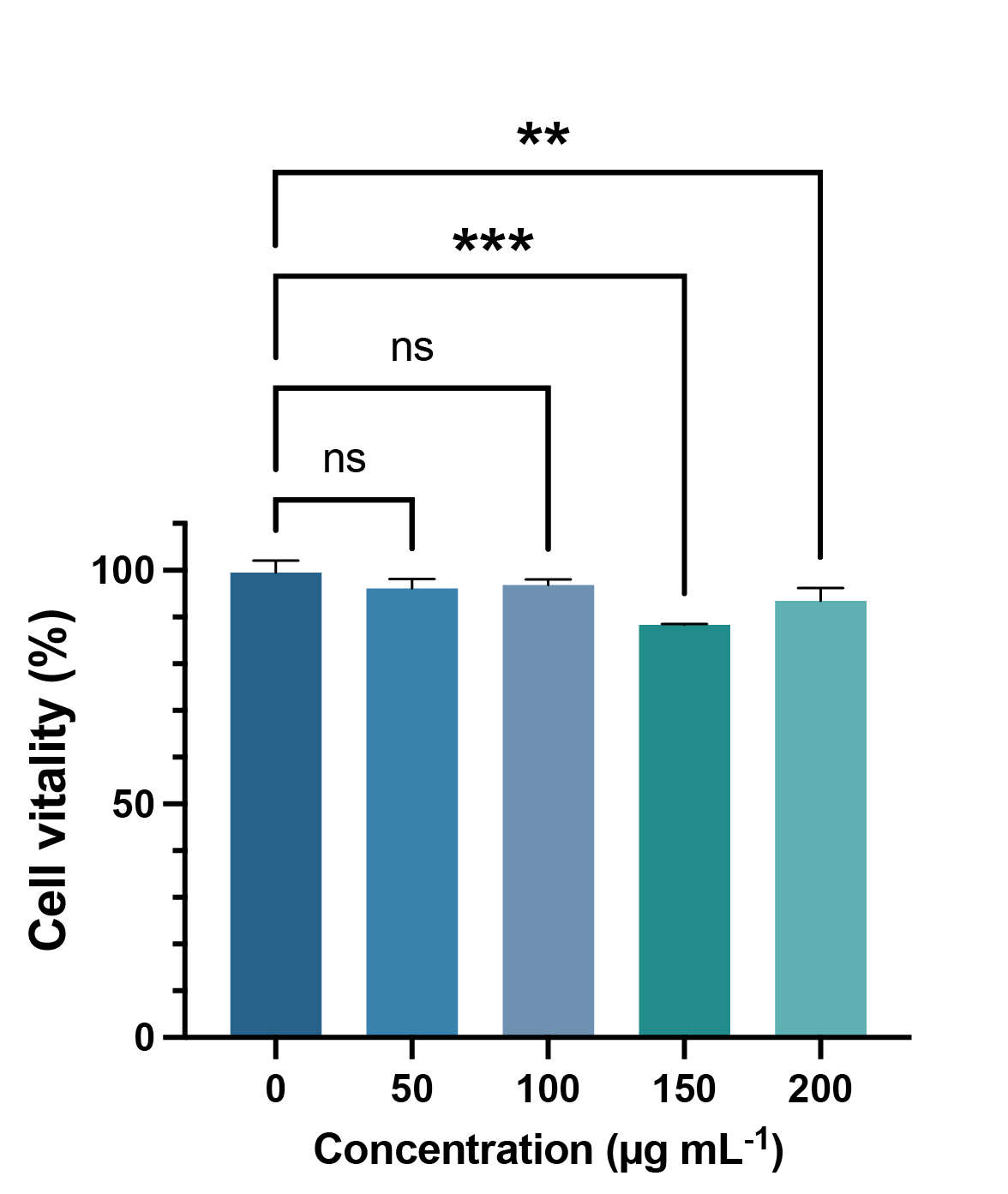


**Figure S7.** *In vitro* cell toxicity experiments. Viability of differentiated 3T3-L1 cells treated with different concentration of hmPDA for 48 hours.


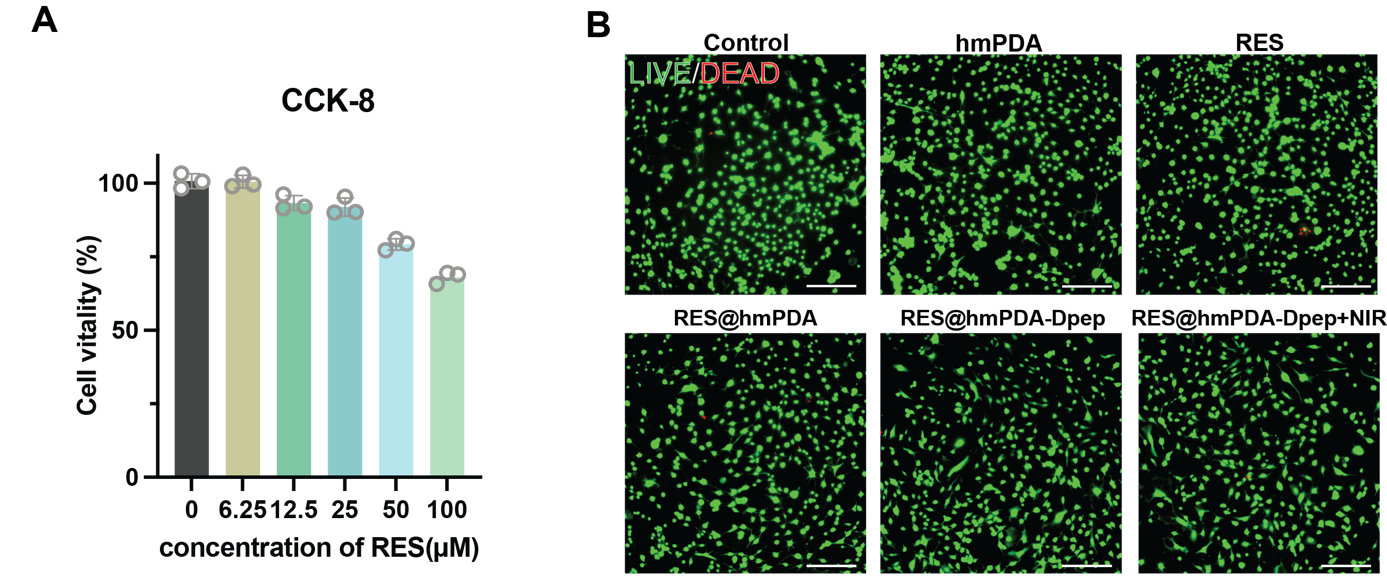


**Figure S8.** *In vitro* cell toxicity experiments. (**A**) Viability of differentiated 3T3-L1 cells treated with different concentration of resveratrol for 48 hours. (**B**) Live-dead cell staining of 3T3-L1 cells after exposing to different treatment. Scale bar = 300 μm.


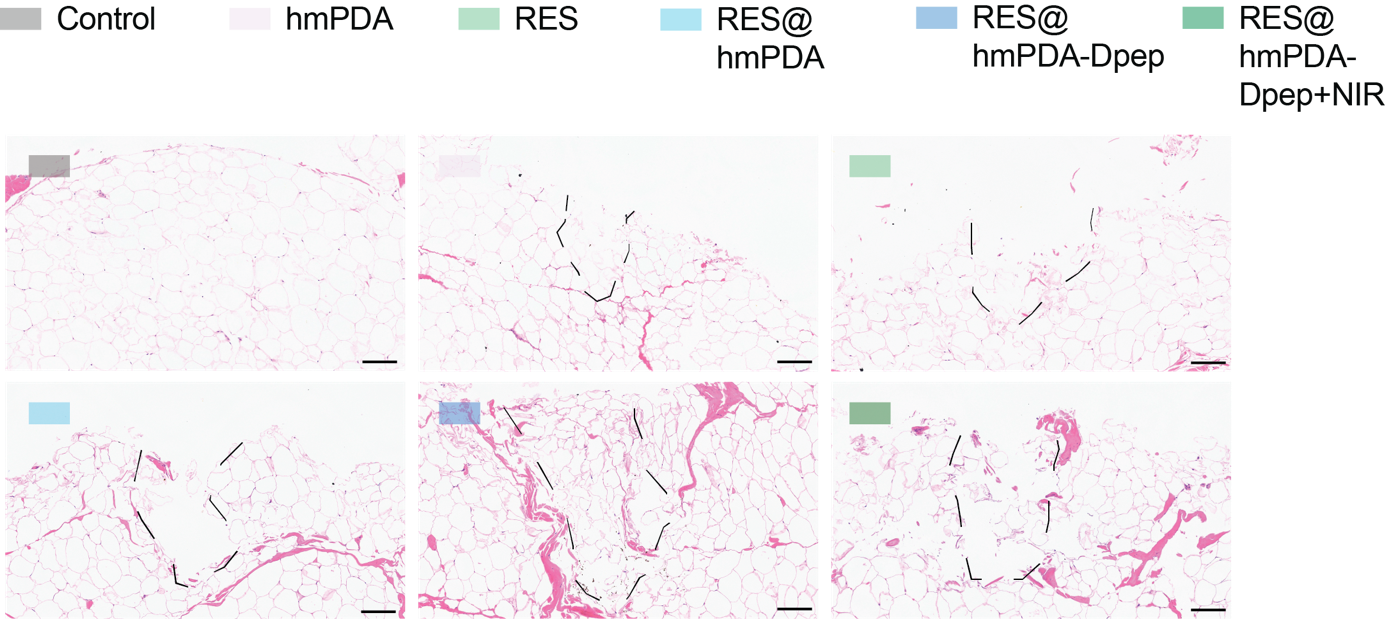


**Figure S9.** H&E staining showed punctures in porcine subcutaneous adipose tissue after being penetrated by microneedles. Scale bar = 300 μm.


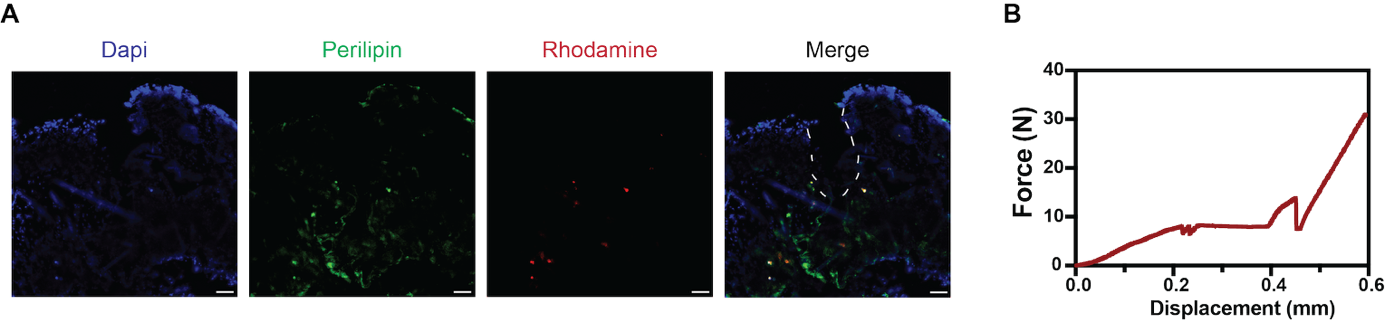


**Figure S10.** (**A**) Representative fluorescence micrographs of skin cross-sections collected after microneedle insertion. Nuclei were counterstained with DAPI (blue). Adipocytes were identified by immunostaining for perilipin-1 (green). Rhodamine-labeled nanoparticles are shown in red, indicating the distribution of the nanocarrier delivered by the microneedle patch. The merged image demonstrates localization of rhodamine signals adjacent to the perilipin-positive adipose compartment (dashed outline). Scale bar = 100 μm. (**B**) Compression force–displacement curve of the dissolving microneedle patch.


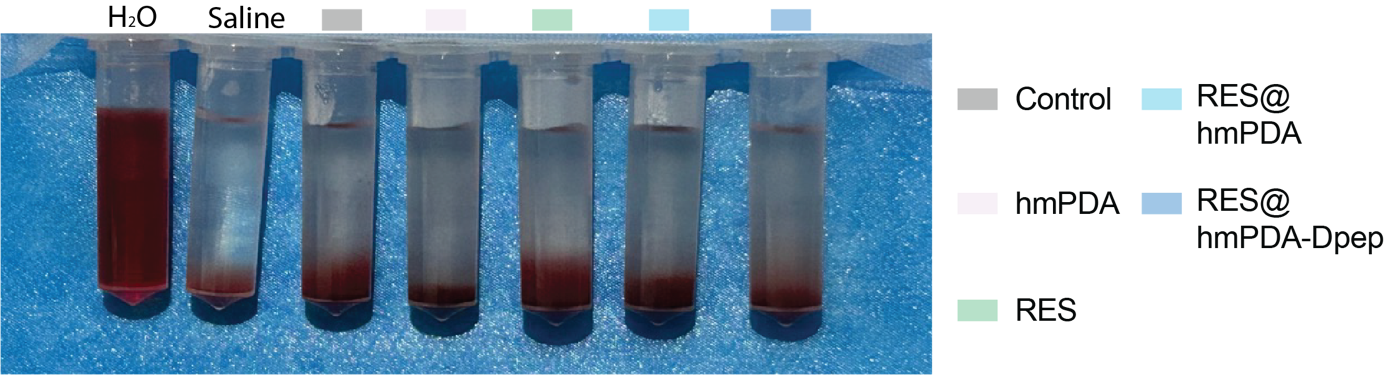


**Figure S11.** Optical images for hemolysis test of different microneedle groups.


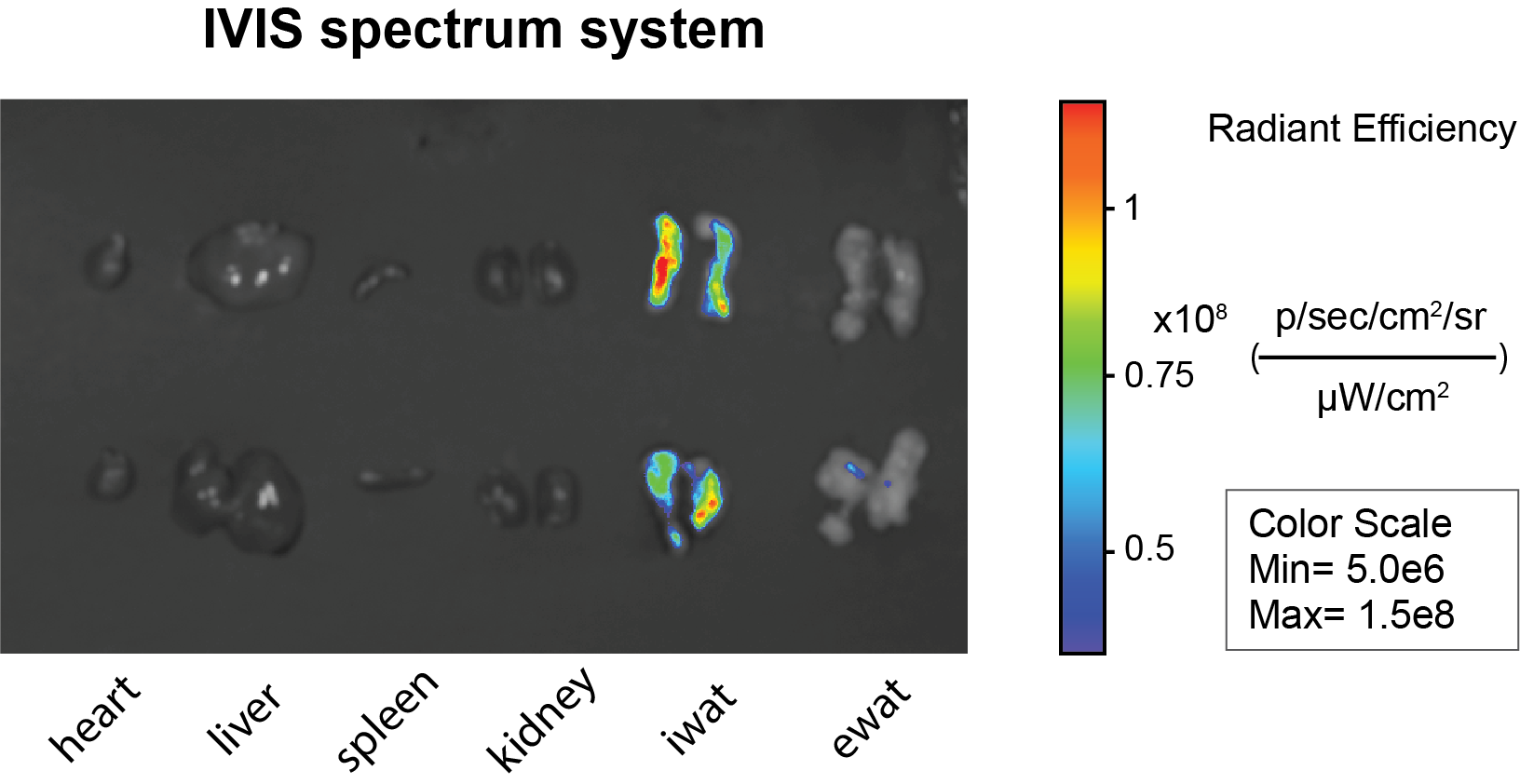


**Figure S12. Ex vivo fluorescence imaging of tissue samples after microneedle insertion.** Representative epi-fluorescence images acquired using an IVIS Spectrum system. Tissues collected **24 h** after microneedle insertion are shown in the **top row,** and tissues collected **48 h** after insertion are shown in the **bottom row.**


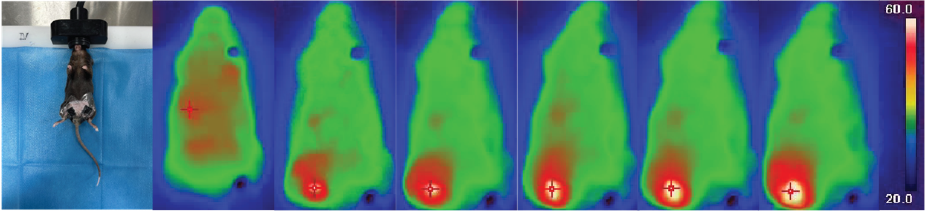


**Figure S13.** Thermal images of diet induced obese (DIO) mice treating with photothermal browning therapy.
